# ANDRO-IVF: a novel protocol for poor responders to IVF controlled ovarian stimulation

**DOI:** 10.5935/1518-0557.20180011

**Published:** 2018

**Authors:** Ludmila Bercaire, Sara MB Nogueira, Priscila CM Lima, Vanessa R Alves, Nilka Donadio, Artur Dzik, Mario Cavagna, Renato Fanchin

**Affiliations:** 1 IVF Centre - Hospital Pérola Byington, São Paulo - SP, Brazil; 2 IVF Centre, Hospital Foch, Suresnes, France

**Keywords:** Fertilization *in vitro*, androgens, ovulation induction, oocyte retrieval, fertility agents, primary ovarian insufficiency

## Abstract

**Objective:**

This study aimed to assess a novel protocol designed to improve poor ovarian response through intra-ovarian androgenization. The endpoints were: number of oocytes and mature oocytes retrieved, fertilization, cancellation and pregnancy rates.

**Methods:**

This prospective crossover study enrolled poor responders from previous ovarian stimulation cycles submitted to a novel protocol called ANDRO-IVF. The protocol included pretreatment with transdermal AndroGel(r) (Besins) 25 mg, oral letrozole 2.5 mg and subcutaneous hCG 2500 IU; cycle control was performed with estradiol valerate and micronized progesterone; ovarian stimulation was attained with gonadotropins FSH/LH 450 IU, GnRH antagonist and hCG 5000 IU.

**Results:**

Fourteen poor responders were enrolled. One patient did not meet the inclusion criteria. Thirteen patients previously summited to the standard protocol were offered the ANDRO-IVF Protocol.-Standard Protocol: Mean age: 35.30 years; cancellation rate: 61.53%; mean number of MII oocytes retrieved per patient: 1.8; fertilization rate: 33.33%. Only two patients had embryo transfers, and none got pregnant.-ANDRO-IVF Protocol: Mean age: 35.83 years; cancellation rate: 7.69%; mean number of oocytes retrieved per patient: 5.58, MII oocytes: 3.91. ICSI was performed in 84.61% of the patients and a mean of 1.5 embryos were transferred per patient. Fertilization rate: 62.5%; cumulative pregnancy rate: 16.66%; mean duration of stimulation: 9.77 days.

**Conclusion:**

ANDRO-IVF allows intra-ovarian androgenization by increasing serum and intra-follicular androgen levels and preventing androgen aromatization. This protocol apparently improved clinical outcomes of poor responders in parameters such as number of oocytes retrieved and clinical pregnancy rates. Further randomized controlled trials are needed to confirm these findings.

## INTRODUCTION

Poor ovarian response after stimulation for *in vitro* fertilization (IVF) is still one of the most important challenges in reproductive medicine. Several adjuvant treatments have been proposed to determine the factors interfering with follicular output ratios and increased ovarian response to gonadotropins, but consensus has not been reached around a strategy to treat poor responders undergoing IVF (Genro *et al*., 2010). Despite the limited data supporting the isolated use of adjuvants such as DHEA, rLH, hCG or letrozole, transdermal testosterone has apparently improved the outcomes of IVF cycles in poor responders (Bosdou *et al*., 2012). The association between increased live birth and pregnancy rates and use of transdermal testosterone has been described in patients with low ovarian response to controlled ovarian stimulation (COS). The authors of these studies also found that lower FSH doses were needed to attain ovarian stimulation (González-Comadran *et al*., 2012; Jeve & Bhandari, 2016). 

The potential mechanisms through which DHEA and testosterone produce increased pregnancy rates are still unclear. Androgens play a key role in steroidogenesis, acting as a substrate in the conversion of androgens into estrogens through aromatization (Ryan *et al*., 1968). Previous studies in nonhumans described greater numbers of primary and preantral follicles and increased androgen receptor expression (Vendola *et al*., 1998; Hillier *et al*., 1997; Hild-Petito *et al*., 1991). Increased ovarian levels of FSH receptors have also been associated to testosterone administration in animal models (Weil *et al*., 1999). Studies in humans have been limited to small, uncontrolled RCTs with preliminary data, and there is no consensus in the literature about the clinical use of transdermal testosterone in poor responders undergoing IVF (Balasch *et al*., 2006; Fábregues *et al*., 2009). Different protocols, testosterone doses and pretreatment lengths have been proposed, but there still is no consensus over the best pretreatment scheme.

The following rationale was used in the ANDRO-IVF protocol: Testosterone is a steroid hormone thought to increase ovarian response by positively affecting follicular feedback to gonadotropin stimulation, leading to greater oocyte yield and, therefore, increased conception rates (Nagels *et al*., 2015). According to the two-cell two-gonadotropin theory, androgens are essential for the maintenance of adequate follicular steroidogenesis in humans. Increased levels of androgens in the follicular intracellular environment are thought to stimulate the recruitment of preantral and antral follicles and thus increase folliculogenesis and ovarian response to COS.

Intrafollicular androgenization may also be performed with the co-administration of letrozole, as described by Garcia-Velasco. This author described significantly higher levels of testosterone and androstenedione in the follicular fluid of letrozole-treated patients than in untreated poor responders (Garcia-Velasco *et al*., 2005). In 2012, the same authors described the impact of testosterone and hCG in the positive modulation of FSH receptor expression in human granulosa cells *in vitro* (Garcia-Velasco *et al*., 2012). This study aimed to propose and assess a new protocol to improve the ovarian response of poor responders based on three modes of intra-ovarian androgenization (letrozole, transdermal testosterone and hCG).

The primary endpoints were number of oocytes and mature oocytes retrieved from ANDRO-IVF cycles. The secondary endpoints were the cancellation, fertilization, and cumulative pregnancy rates obtained with the protocol. 

## MATERIAL AND METHODS

This prospective crossover study included patients seen between January and October of 2016 at the IVF Center of the Pérola Byington Hospital in São Paulo, Brazil. All poor responders submitted to ovarian stimulation were recruited and invited to join the study. 

The Bologna criteria for poor ovarian response (Ferraretti *et al.,* 2011) were used as inclusion criteria. At least two of the following features had to be present:

- Patients submitted to COS for IVF with <3 oocytes collected or whose COS was cancelled due to lack of response from conventional COS for IVF;
- Patients with low ovarian reserve: Antral follicle count <5-7 and/or antimullerian hormone <1.2;
- Female patients aged >40 years.

The patients enrolled in the study had had IVF cycles with three of fewer oocytes retrieved and antral follicle counts of six or fewer. 

Patients with Müllerian duct anomalies and history of pelvic surgery were excluded.

The individuals included in the study were submitted to a novel protocol called ANDRO-IVF. Their results from the standard protocol were compared to the outcomes produced with the ANDRO-IVF Protocol.

A) Standard Protocol was performed with gonadotropins (FSH+LH) 300 IU up to day 2 or 3; a GnRH antagonist 0.25 mg was administered after a follicle reached the size of 14 mm; and ovulation was triggered with hCG 5000 IU when at least one follicle reached the size of 18-20 mm.

B) The ANDRO-IVF protocol included two treatment phases, as described below:

- Phase 1: ovarian preparation (previous cycle).
- Phase 2: ovarian stimulation (IVF cycle).

The phase of ovarian preparation included intra-ovarian androgenization and cycle control. Androgenization was performed with the application of transdermal testosterone gel AndroGel(r) (Besins Healthcare, São Paulo, Brazil) 25 mg every other day, starting on the first day of the menstrual cycle. Letrozole 2.5 mg was administered orally on a daily basis, and patients were given hCG 2500 IU subcutaneously twice a week. Cycle control was performed with the administration of estradiol valerate 8 mg daily from Day 3 to Day 14 of the menstrual cycle, followed by estradiol valerate 4 mg daily up to Day 15. Micronized progesterone 400mg was given from Day 15 to Day 24 and suspended to promote a new menstrual cycle, in which stimulation occurred.

Ovarian stimulation was performed with recombinant or urinary follicle-stimulating hormone 450 UI FSH/LH. A GnRH antagonist (cetrorelix acetate or ganirelix acetate 0.25 mg/day) was given up to Day 6 of stimulation or when at least one follicle reached a diameter >14 mm. 

Ovulation was triggered with human chorionic gonadotropin hCG 5000 IU after at least one follicle had a diameter >18-20mm diameter.

## RESULTS

Fourteen poor-responders were initially included in the study. One of the patients did not meet the inclusion criteria and was referred to intrauterine insemination. The thirteen patients selected had previously undergone standard IVF cycles and were submitted to the ANDRO-IVF protocol. The possible differences between procedures were analyzed with the aid of the chi-square test. The results are shown in Table 1.

**Table 1:** Results

Results	Standard Protocol	ANDRO-IVF Protocol	Sig. (*p*)
Mean age	35.30	35.83	-
Number of cycles	13.00	13.00	-
Cancellation rate/cycle	61.54%	7.69%	<0.001
Mean number of oocytes/patient	2.00	5.58	<0.001
Mean number of MII oocytes/patient	1.80	3.92	0.002
Fertilization rate/patient	33.33%	62.50%	0.040
Mean number of embryos/patient	0.60	1.50	0.017
Cumulative pregnancy rate/patient	0/5 (0%)	2/12 (16.67%)	-
Mean duration of stimulation (days)	7.72	9.77	-

### Standard IVF cycles

The mean age of the patients at the time of IVF was 35.30 years. Thirteen patients had a total of 13 cycles, eight of which cancelled (61.53%) for lack of response. Ten oocytes were retrieved from the five patients submitted to standard IVF, yielding a mean of two oocytes per cycle. Nine were mature oocytes and each patient had a mean of 1.8 MII oocytes. ICSI was performed in five of the 13 patients (38.46%). The fertilization rate was 33.33% (3/9). Three oocytes developed into embryos (0.6 embryo/patient) and two were transferred, but none of the patients became pregnant. The mean duration of stimulation was 7.72 days.

### ANDRO-IVF Protocol Cycles

The same thirteen patients were submitted to the ANDRO-IVF Protocol. Their mean age at the time of the second procedure was 35.83 years. Only one patient had her cycle cancelled due to lack of response, yielding a cancellation rate of 7.69%. The mean follicle count was 4.58. The mean number of oocytes retrieved and of mature oocytes was 5.58 and 3.91, respectively. ICSI was performed in eleven of the thirteen patients (84.61%) and 18 embryos were obtained (1.5 embryo/patient). The fertilization rate was 62.5%. Two patients became pregnant, one after a fresh embryo transfer and the other after a frozen embryo transfer. The cumulative pregnancy rate was 16.66% (two pregnancies/12 embryo transfers). The mean duration of stimulation was 9.77 days.

## DISCUSSION

Previous studies have shown that androgens stimulate the proliferation of granulosa cells and the growth of larger follicles in the ovaries of nonhuman primates (Vendola *et al*., 1998). The authors showed that androgens increased the number of primary, small and medium-sized antral follicles in the ovaries of rhesus monkeys. They also observed that androgens increased IGF-I expression in primordial follicle oocytes. Increased androgen receptor expression has been observed in the granulosa cells of growing follicles, mainly in pre antral and early antral follicles (Hillier *et al.,* 1997; Hild-Petito *et al*., 1991). The authors also found that androgen-treated monkeys had increased growth of secondary and small antral follicles, and increased proliferation and decreased apoptosis of granulosa cells. 

Several clinical trials investigated the role of androgen administration to improve ovarian response in poor responders undergoing IVF. Although some studies did not support androgen supplementation to improve live birth rates (Sunkara *et al*., 2011), a recent meta-analysis showed increased clinical pregnancy and live birth rates in poor responders given transdermal testosterone (Bosdou *et al.,* 2012). Another Cochrane meta-analysis included 17 randomized clinical trials and 1496 patients, mostly poor responders undergoing standard IVF. The authors found that pretreatment with testosterone correlated with higher live birth rates when compared to placebo or no treatment (OR 2.60, 95% CI 1.30 to 5.20). Women with an eight percent chance of achieving a live birth if treated with placebo or not offered treatment saw their chances rise to 10-32% (Nagels *et al.,* 2015). Clinical studies also revealed that 25% of clinics worldwide have adopted co-treatment with androgens for this group of patients (Fouany & Sharara, 2013).

Recent experimental studies looking into the intracellular environment have assessed the paracrine regulation of steroidogenesis in theca cells by co-culturing theca and granulosa cells, and the results indicated increased steroidogenesis in theca cells. These models might answer questions involving the hormone interactions affecting ovarian stimulation protocols (Liu *et al.,* 2015).

A prospective case-control study from a respected IVF center compared the intrafollicular and serum concentrations of LH, estradiol (E_2_), progesterone, testosterone, and androstenedione of patients showing poor response to treatment and egg donors with normal response (de los Santos *et al*., 2013). They observed that LH and androgen secretion in preovulatory follicles was similar in both groups, suggesting that the problem was related to poor response to COS in spite of normal levels of follicular hormones. They suggested that long-interval androgen pretreatment might potentially increase the recruitment of small pre antral and antral follicles rather than increasing the intrafollicular androgen levels in IVF cycles.

Our findings were similar to the reports of a prospective therapeutic self-controlled clinical trial that included 25 patients whose first and second IVF cycles were cancelled due to poor follicular response despite normal baseline FSH levels (Balasch *et al*., 2006). The authors described a greater than fivefold increase in the number of recruited follicles in 80% of the patients, a mean of 5.8±0.4 oocytes produced, a cancellation rate of 20%, and a clinical pregnancy rate of 30% per oocyte retrieval. They concluded that pretreatment with transdermal testosterone might be useful for poor responders with normal baseline FSH levels.

A previous RCT enrolled 62 poor responders with cancelled cycles in the first attempt at IFV and randomized them in two groups, one given pretreatment with transdermal testosterone and the other treated with high-dose gonadotropins in a mini-dose GnRH agonist protocol. The proportion of poor response cycles was significantly lower in the testosterone group (32.2%) than in the high-dose gonadotropin group (71%, IC 95% 15.7 - 61.6; *p*=0.05). The findings suggested that transdermal testosterone pretreatment might improve the ovarian sensitivity to FSH and the follicular response to gonadotropins of poor responders (Fábregues *et al.,* 2009). 

Another recent study described the advantages of administering transdermal testosterone 12.5 mg for 21 days before IVF. Testosterone pretreatment was associated to lower total doses of r-FSH and shorter length of COS. The number of oocytes, mature oocytes, fertilized oocytes, and good-quality embryos retrieved and clinical pregnancy rates were also significantly higher in the pretreatment group (Kim *et al*., 2011).

In contrast, another RCT did not find beneficial effects connected with administering 10 mg of testosterone daily before IVF (Massin *et al.,* 2006). The divergence between the studies may have been caused by the differences in testosterone doses, administration regimes or inclusion criteria, and the fact that the latter enrolled not only women meeting the Bologna criteria, but also female patients with possibly suboptimal response to COS.

A recent meta-analysis initially including 10 papers had to cut the number of papers down to three because of the large heterogeneity between the studies and androgen administration protocols. The authors concluded that pretreatment with transdermal testosterone prior to IVF might improve the clinical outcomes of poor responders, but indicated that the results be analyzed with caution due to the lack of larger and more robust RCTs.

Our protocol fosters intra-ovarian androgenization using different mechanisms: first, androgen serum levels are increased with the administration of transdermal testosterone gel every other day; then, intrafollicular androgen levels are increased with the administration of hCG twice a week; and finally, androgen aromatization to estrogen is prevented with the daily administration of an aromatase inhibitor, letrozole. Letrozole increases the intra-ovarian concentration of androgens by inhibiting aromatase activity (Garcia-Velasco *et al*., 2005). Oral estradiol and vaginal progesterone were administered to control the menstrual cycle. The pregnancy rates achieved in our study were similar to the rates published in previous studies. Considering that pregnancy rates among poor responders is close to or lower than 10%, our results suggested that this novel protocol might be a valid alternative to treat poor responders.

## CONCLUSION

Our protocol seemed to improve clinical outcomes in poor responders in parameters such as number of oocytes and clinical pregnancy rates. Further RCT studies are needed to confirm these findings.
